# Identifying Oxysterols Associated With Age and Diet in Mice Using Optimized Reversed‐phase Liquid Chromatography‐Mass Spectrometry (RPLC‐MS)

**DOI:** 10.1002/jssc.70274

**Published:** 2025-09-29

**Authors:** Indhumathy Subramaniyan, Benjamin Barr, Ninh M. La‐Beck, Benjamin G. Janesko, Lauren Gollahon, Li Li

**Affiliations:** ^1^ Clinical Pharmacology and Experimental Therapeutics Center Jerry H. Hodge School of Pharmacy Texas Tech University Health Sciences Center Dallas Texas USA; ^2^ Department of Biological Sciences Texas Tech University Lubbock Texas USA; ^3^ Department of Immunotherapeutics and Biotechnology Texas Tech University Health Sciences Center, Jerry H. Hodge School of Pharmacy Abilene Texas USA; ^4^ Department of Chemistry & Biochemistry Texas Christian University Fort Worth Texas USA

## Abstract

Structurally similar oxysterols such as 7α‐hydroxycholesterol, 7β‐hydroxycholesterol, and 7‐ketocholesterol; 5,6α‐ and 5,6β‐epoxycholesterol; and 24(*R*/*S*)‐hydroxy cholesterol, 25‐hydroxy cholesterol, and 27‐hydroxycholesterol are traditionally difficult to resolve using reversed‐phase liquid chromatography (RPLC). We present a simple yet highly optimized method for the simultaneous quantification of eight oxysterols using RPLC coupled with mass spectrometry (MS) without derivatization. Optimal separation of most oxysterols was achieved at a lower column temperature (25°C), with specific combinations of stationary and mobile phases enhancing resolution, particularly for isomeric pairs such as 7α‐/7β‐OHC, 5,6α‐/5,6β‐EC, 24 R/S‐OHC, and 25‐OHC. Although certain analytes (e.g., 24*S*‐OHC and 27‐OHC) remained challenging to separate due to similar retention behavior, they were distinguishable by their unique MRM transitions. We applied this method to investigate oxysterol changes in a longitudinal mouse study comparing a normal diet to a high‐fat diet. Liver and brain samples were analyzed, revealing distinct distribution patterns between the two organs. Notably, 24(*S*)‐hydroxycholesterol levels, a signature cholesterol metabolite exclusively produced in the brain, increased with age independent of diet. In contrast, 5,6α‐epoxycholesterol production in the liver was influenced by both age and dietary factors. Our method provides a robust tool for studying oxysterol variation and its implications in aging and diet, offering new insights into cholesterol‐derived lipid regulation across different physiological conditions.

## 1 Introduction

Oxysterols are oxidized derivatives of cholesterol that play crucial roles in cholesterol metabolism, signaling, and disease pathology [1, 2]. Oxysterols can be formed enzymatically or through autooxidation from cholesterol. For example, 7α‐hydroxycholesterol (7α‐OHC), 7‐ketocholesterol (7‐KC), 5,6 α‐epoxycholesterol (5,6α‐EC), and 25‐hydroxycholesterol (25‐OHC) can be produced through both pathways. 7β‐hydroxycholesterol (7β‐OHC) and 5,6β‐epoxycholesterol (5,6β‐EC) are produced primarily from autooxidation. Some oxysterols are organ‐specific: 24*S*‐OHC is produced almost exclusively in the brain enzymatically, while 27‐OHC is primarily generated enzymatically in liver [3]. The liver plays a central role in regulating oxysterol levels by both producing and eliminating them through bile. 24(*S*)‐hydroxycholesterol (24*S*‐OHC) and 27‐hydroxycholesterol (27‐OHC) can also diffuse across the blood–brain barrier (BBB) and influence brain cholesterol synthesis, highlighting their role in the liver–brain axis [4].

Isomers such as 7α‐/7β‐OHC, 5,6α/β‐EC, and 24 (*R*, *S*)‐/25‐/27‐OHC share very similar structures, but exhibit different biological effects. 7α‐OHC is a key intermediate in cholesterol catabolism and bile acid synthesis, maintaining lipid homeostasis. Dysregulation of this pathway is associated with metabolic and cardiovascular diseases [5]. Its isomer 7β‐OHC is primarily linked to oxidative stress and pathological conditions, contributing to inflammation and cytotoxicity. 5,6α‐EC is metabolized into oncosterone, which promotes tumor growth in breast cancer [6]. However, it can also be converted into dendrogenin A (DDA), a tumor suppressor in normal breast tissue. Elevated levels of 5,6α‐EC have been observed in the frontal and occipital cortex of Alzheimer's disease patients [7]. To date, little is known about the specific function of its isomer 5,6β‐EC. While neither diastereomer of 5,6‐EC is classified as a potent carcinogen, their metabolism in mammalian cells has been linked to cancer progression [8]. In contrast, 5,6‐ECs have also demonstrated bioactive properties against human myeloma cells [9]. 24*S*‐OHC is the major cholesterol metabolite in the brain, synthesized via CYP46A1, an enzyme expressed predominantly in neurons. The circulating levels of 24*S*‐OHC depend on cerebral production and hepatic clearance, making it an important marker of brain cholesterol metabolism [10, 11, 12]. Its isomer 24(*R*)‐OHC has not been identified naturally. Its other isomer 27‐OHC, is involved in sterol transport from peripheral tissues to the liver and serves as a substrate for bile acid synthesis. Abnormal levels of 27‐OHC are implicated in atherosclerosis, tumor progression, and breast cancer metastasis through immune modulation, as well as neurological disorders [1, 13, 14, 15].

Separation of structural isomers such as 7α‐/7β‐OHC, 5,6α‐/EC, and 24(*R*/*S*)‐, 25‐, and 27‐OHC remains a significant analytical challenge due to their identical molecular weights and similar fragmentation patterns in multiple reaction monitoring (MRM) mode [16, 17]. Numerous analytical approaches have been developed for accurate quantification of different oxysterol groups. Among them, gas chromatography (GC) with derivatization has been widely employed [18, 19]. However, derivatization adds complexity and time to sample preparation. Therefore, the development of derivatization‐free methods using widely available reversed‐phase liquid chromatography (RPLC) is of considerable interest, as it can enhance both efficiency and accessibility. Comprehensive chromatographic behavior of 32 oxysterols has been demonstrated using GC, normal‐phase HPLC, and chiral separations, as well as reversed‐phase HPLC, including Alltima 5‐µm C18 (250 mm × 4.6 mm i.d., Alltech) and Dynamax 8‐µm C8 columns (250 mm × 4.6 mm i.d., Varian, Walnut Creek, CA) in various solvent systems [17, 23]. In addition, separation of different oxysterol groups has been reported using a variety of RPLC columns, such as the Restek Raptor Inert Biphenyl (2.1 × 100 mm, 1.8 µm), the modified C18 L‐column2 ODS (2 µm, 2.1 mm × 150 mm) [20], and the Phenomenex Gemini C18 (250 mm × 4.6 mm, 5 µm) [21]. Despite all these attempts, previous methods were generally limited to separating oxysterols with distinct MRM transitions or, at best, resolving a single pair of isomers. Simultaneous separation of the most structurally challenging isomers—such as 7α‐/7β‐OHC, 5,6α‐/5,6β‐EC, and 24(*R*/*S*)‐, 25‐, and 27‐OHC—has not been achieved.

**FIGURE 1:**
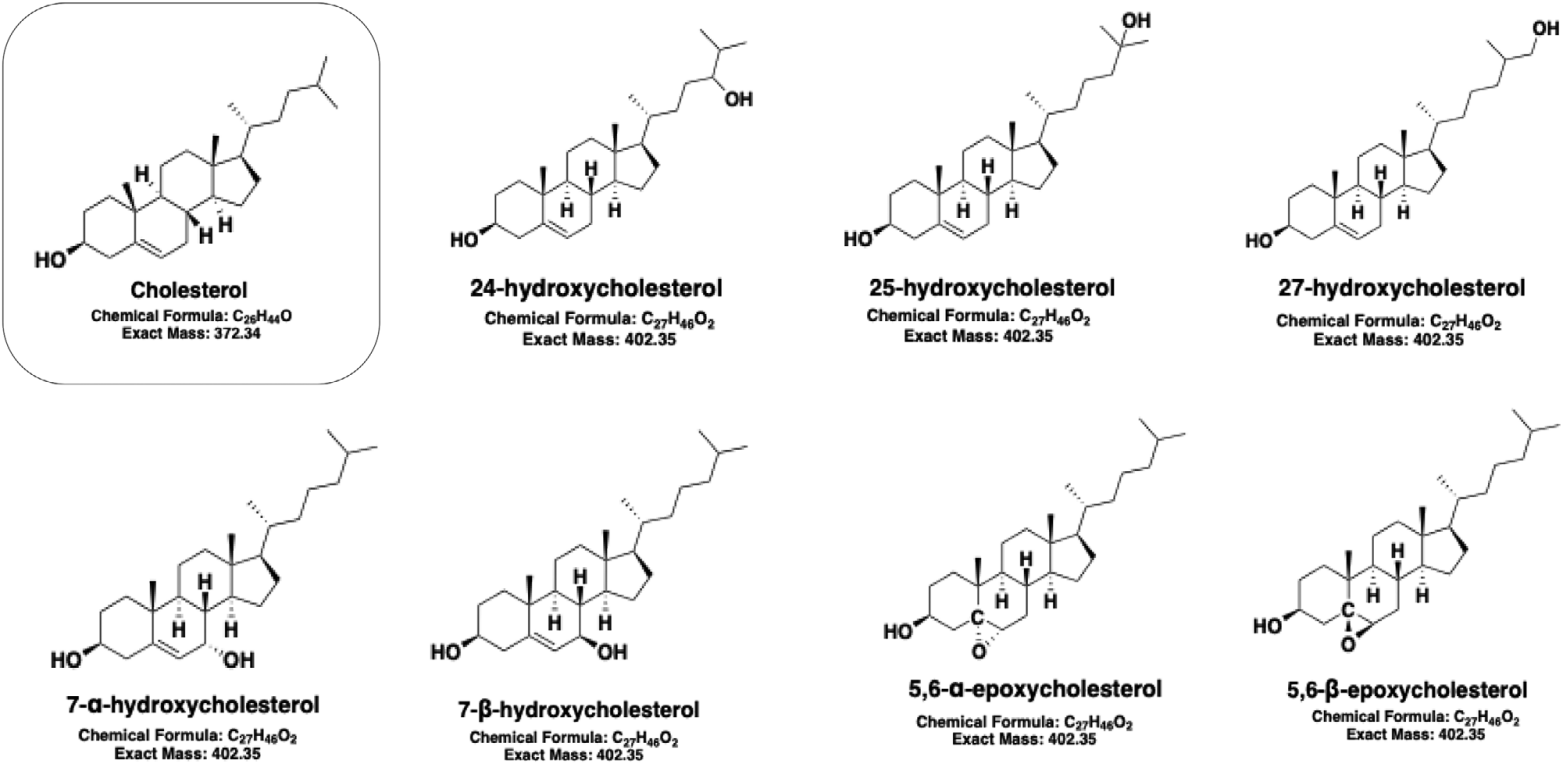
Structures of cholesterol and its oxysterol isomers.

In this study, we aimed to separate structurally similar oxysterol isomers that present significant challenges in RPLC–mass spectrometry analysis at low temperature to achieve better separation as well as to reduce autooxidation of cholesterol to oxysterols on Acquity UPLC BEH C18 or C8 columns. Among the eight oxysterols selected for analysis, seven shared the same MRM transition (*m*/*z* 385.4→367.5) (see Figure 1), complicating their identification and quantification. Most oxysterol ions were detected as [M‐H_2_O+H]⁺, indicating the loss of one water molecule from their molecular weight (Table S1). Notably, isomeric pairs such as 7α‐/7β‐OHC, 5,6α‐/EC, and side chain oxidized species, including 24*R*‐, 24*S*‐, 25‐, and 27‐OHC, were particularly difficult to resolve using standard RPLC methods. We demonstrate here that baseline separation of these challenging isomers, despite their shared MRM transitions, can be achieved using optimized RPLC–MS conditions. This method development enables more accurate and confident identification and quantification of oxysterols in complex biological samples.

## 2 Materials and Methods

### 2.1 Chemicals and Reagents

Optima LC‐MS grade acetonitrile (ACN), methanol (MeOH), water, and formic acid were purchased from Fisher Scientific (Hampton, NH). Cholesterol, 7α‐hydroxycholesterol, 7β‐OHC, 7‐KC, 5,6α;‐epoxycholesterol, 5,6β‐EC, 24*R*‐hydroxycholesterol, 24*S*‐hydroxycholesterol, and 25‐OHC, were obtained from Cayman Chemicals (Ann Arbor, MI); 27‐OHC was obtained from Enzo Life Sciences Inc.

Isotope‐labeled internal standards (IS), including d7 forms of cholesterol, 5,6α;‐epoxycholesterol, 5,6β‐EC, 22(*S*)‐hydroxycholesterol, and the d6 form of 27‐OHC, were purchased from Avanti Research (Alabaster, AL). The d7 form of 7α‐hydroxycholesterol, 7β‐OHC, 7‐KC, 24*R*, *S*‐hydroxycholesterol, and the d6 form of 25 hydroxycholesterol were purchased from CDN Isotopes (Quebec, Canada); 24 hydroxycholesterol‐d4 was purchased from LGC Standards Ltd. (UK).

### 2.2 Normal Diet‐Fed Mice and High‐Fat Diet‐Fed Mice

The C3H/HeJ (C3H) mouse strain serves as an alternative model for studying obesity development, particularly in non‐predisposed populations over time. Animal handling followed regulatory compliance under the approved Texas Tech University IACUC protocol number 19021‐02. A total of 64 female C3H mice were obtained at 4 weeks old from Jackson Laboratories (Bar Harbor, ME, USA) and allowed 1 week to acclimate. Mice were housed four per cage under controlled temperature and humidity and in 12 h‐light/dark‐cycle rooms. Half of the mice were then given a high‐fat casein (HFC) diet (46% kcal fat) and the remainder were given a reference (CC) diet (11% kcal fat), for up to 18 months. Diets and water were supplied ad libitum for the duration of the study. Mice were weighed and given assessments of health weekly. At cross‐sectional time points of 6, 12, and 18 months, three mice were sacrificed from each diet group via CO_2_ euthanasia followed by cervical separation. Complete dietary formulations can be found in Barr and Gollahon, as well as findings indicating that the HFC dietary group developed diet‐induced obesity (DIO) over the course of the study [16]. A total of 18 mice from each diet group and three timepoints were used for assessment of oxysterol isomers from their brains and livers.

### 2.3 Chromatographic Conditions

The liquid chromatography system used in this study was a Shimadzu CBM‐20A Nexera X2 series LC system (Shimadzu Corporation, Kyoto, Japan) equipped with a degasser (DGU‐20A), two binary pumps (LC‐30AD), an autosampler (SIL‐30AC), and a column oven (CTO‐30A). The autosampler was maintained at 10°C. Various chromatographic conditions were evaluated by modifying the column type, mobile phase, temperature, and separation gradient to optimize the separation of oxysterols.

When a 150 mm C18 column (Acquity Premier Vanguard FIT BEH 1.7 µm, 150 × 2.1 mm C18) was used, two mobile phase systems were explored: an ACN/water system and an MeOH/water system. ACN/water mobile phase system consisting of mobile phase A (MPA, 0.1% formic acid in water) and mobile phase B (MPB, 0.1% formic acid in ACN). The chromatographic flow rate was set at 0.4 mL/min. The gradient elution program started with 75% of MPB for the first 2 min, then 98% MPB at 15 min maintained until 23 min, then returned to 75% MPB at 23.1 min until the end of the run at 25 min; the MeOH/water mobile phase system consisting of MPA (0.1% formic acid in water), and MPB (0.1% formic acid in MeOH). The chromatographic flow rate was set at 0.3 mL/min. The gradient elution program started with 75% MPB for the first 2 min, 98% MPB at 22 min, and was maintained to 30 min. Then return to 75% MPB at 30.1 min and keep that until the end of the chromatographic run at 33 min.

When a 100 mm C8 column (Acquity UPLC BEH C8 1.7 µm, 100 × 2.1 mm column from Waters) was used, the same mobile phase system components were used as described for the C18 column. In both mobile phase systems, the gradient elution program started with 70% of MPB for 0.5 min, 80% MPB at 8.5 min, and 98% MPB by 9 min and was maintained until 10.5 min, then returned to 70% MPB at 10.6 min and maintained until the end at 12 min. The chromatographic flow rate was set at 0.4 mL/min.

10 µL oxysterol samples and 5 µL cholesterol samples were injected in all experiments. Column temperatures at 25°C, 40°C, and 55°C were evaluated.

### 2.4 Mass Spectrometry Conditions

The mass spectrometric system consisted of a SCIEX QTRAP 6500+ mass spectrometer (Redwood, CA, USA) equipped with a Turboionspray ionization source. Quantitative mass spectrometric conditions equipped with positive ion electrospray ionization in MS/MS detection. The gas parameters included nebulizer gas at 50 psi, auxiliary gas at 60 psi, curtain gas at 45 psi, and CAD gas set to medium. The ion spray voltage of the source was set to 5500 V, and the temperature was set to 550°C. The compound‐specific parameters were listed in Table S1. Detection of the ions was carried out in the MRM mode. The analytical data obtained was processed by Analyst software (version 1.7.3).

### 2.5 Sample Preparation for Mass Spectrometry Analysis

Liver and brain tissues were lysed in Pierce IP lysis buffer supplemented with protease inhibitor (one Roche cocktail tablet per 10 mL buffer) and butylated hydroxytoluene (5 mg/mL) in DMSO at a tissue‐to‐buffer ratio of 100 mg: 500 µL. The mixture was homogenized using a mechanical probe in a 2.0 mL round‐bottom centrifuge tube for 30 s at 10 000 rpm.

Cholesterol and oxysterol primary stocks were made in chloroform at a concentration of 1 mg/mL, stored at −80°C. All working stock solutions and further dilutions were also made in MeOH and stored at −20°C and used within 15 days. Isotopically labeled IS stock solutions were prepared and handled in similar fashion. The calibration curve standards as well as quality control (QC) standards were prepared in MeOH with 0.1% formic acid at concentrations of 1, 5, 10, 50, 100, 250, 500, and 1000 ng/mL for each oxysterol, and 0.1, 0.5, 1, 5, 10, 25, 50, and 100 µg/mL was used for cholesterol. The concentrations of low‐quality control (LQC) standard, medium‐quality control (MQC) standard, and high‐quality control (HQC) were prepared at 15, 150, and 600 ng/mL for each oxysterol and were prepared at 1.5, 15, and 150 µg/mL for cholesterol.

Each 85 µL CC/QC sample or 85 µL tissue homogenate was spiked with 10 µL of IS mix (500 ng/mL d7/d6 each labelled oxysterol and 50 µg/mL for cholesterol d7) and 5 µL BHT (100 mg/mL in DMSO). Then 100 µL of LC‐MS grade MeOH and 200 µL of dichloromethane (DCM) were added to this and thoroughly mixed on the benchtop vortex mixer for 1 min. 100 µL of LC‐MS grade water was added to the mixture and vortexed for 10 s, then the glass tubes were kept at room temperature for 15 min for phase separation. Then the samples were centrifuged at 4000 × *g*, 4°C for 10 min. Finally, 170 µL of the lower DCM layer was collected and dried in the speed vacuum and reconstituted in 100 µL of MeOH 0.1% formic acid was added to reconstitute the extracts for LC‐MS analysis.

### 2.6 Computational Methods

Calculations model the relative retention times (RT) of oxysterols in terms of predicted hexane:water partition coefficients logP_hw_. Calculations combine conformational search and density functional theory (DFT) refinement of stable structures [17]. Conformational searches use the CREST metadynamics and molecular dynamics algorithm for automated exploration of conformational space [18], combined with the GFN2‐xTB tight‐binding Hamiltonian [19] and the GBSA continuum solvent models for hexane and water [20]. For each molecule, we perform two CREST conformational analyses, one in water and one in hexane. The three lowest‐energy structures are refined using DFT calculations in the Gaussian 16 package [21], employing the ωB97X‐D dispersion‐corrected DFT functional [22], the def2tzvp basis set [23], the SMD continuum models for hexane and water solvent [24], and ωB97X‐D/6‐31G(d) geometries and vibrational frequencies. Gibbs free energies are taken as the free‐particle‐rigid‐rotor‐harmonic‐oscillator (RRHO) free energy correction computed in continuum solvent [25, 26].

## 3 Result and Discussion

### 3.1 Chromatography Optimization

#### 3.1.1 Selectivity of C18 Versus C8 Columns

Reversed‐phase high‐performance liquid chromatography (RP‐HPLC) relies on hydrophobic stationary phases, typically composed of alkyl chains. C18 columns, with longer chains, exhibit greater hydrophobicity compared to C8 columns, which can influence separation efficiency and selectivity. While analytes generally elute in order of increasing hydrophobicity, oxysterol isomers often exhibit unexpected selectivity differences between C18 and C8 columns.

**FIGURE 2:**
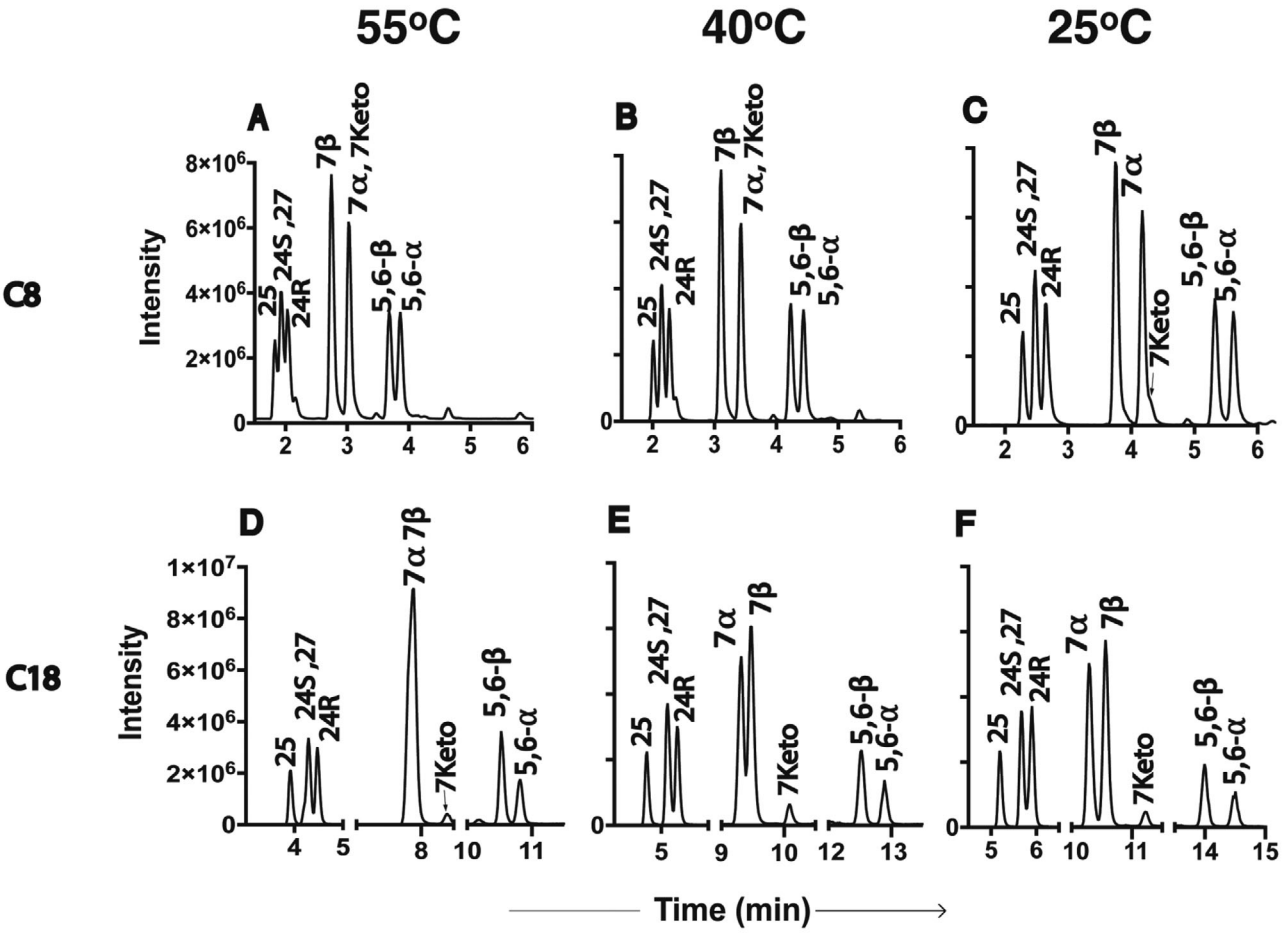
Separation of oxysterols using an acetonitrile mobile phase under different column types and temperatures.

**FIGURE 3:**
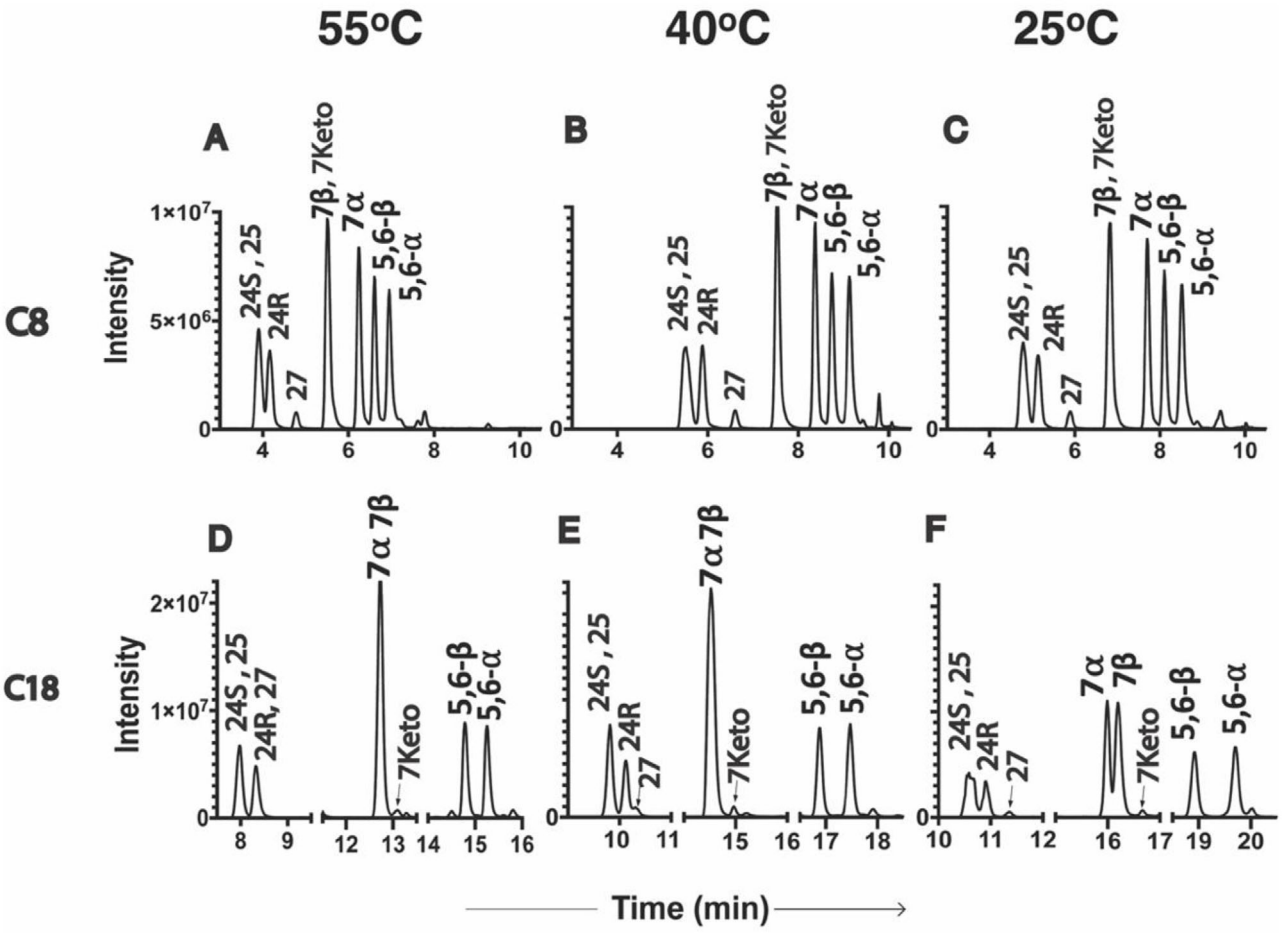
Separation of oxysterols using a methanol mobile phase under different column types and temperatures.

In our experiments, we observed differing selectivity for isomer pairs on C8 (Figures 2A–C and 3A–C) and C18 (Figures 2D–F and 3D–F) columns, particularly for 7α‐/7β‐OHC and 5,6α‐/5,6β‐EC. Regardless of the mobile phase used, ACN (Figure 2A–F) or MeOH (Figure 3A–F), the 7α‐/7β‐OHC pair, which coelutes on the C18 column, achieved baseline separation on the C8 column. Interestingly, the elution order of these isomers was reversed: 7β eluted first on the C8 column, whereas 7α eluted first on the C18 column. In contrast, the 5,6α‐/5,6β‐EC pair showed improved separation on the C18 column but remained unresolved on the C8 column (Figures 2A–F and 3A–F). DFT‐predicted hexane:water partition coefficients (logP_HW_, Table S2) are 0.52 log units more positive for 7α‐OHC versus 7β‐OHC and 0.04 log units more positive for 5,6β‐EC versus 5,6α‐EC, consistent with 7α and 5,6β preferring the mobile phase and eluting first on the C18 column. For the remaining four oxysterols‐24*R*‐OHC, 24*S*‐OHC, 25‐OHC, and 27‐OHC—the C18 column demonstrated higher separation efficiency when ACN was used as the mobile phase (Figure 2D–F). The C8 column offered slightly better resolution when MeOH was used (Figure 3A–C). Overall, the separation efficiency was influenced by a combination of factors, including the type of column, mobile phase composition, and column temperature.

#### 3.1.2 Column Temperature

Column separation temperature has a large effect on retention, selectivity, and column efficiency and has long been accepted as an important parameter in liquid chromatography (LC). Despite this fact, temperature optimization has not been very actively utilized in the past. The temperature impact on analyte retention and selectivity differs with each analyte and depends on the properties of the mobile and stationary phases. The relative RTs of 7α‐OHC and 7β‐OHC are highly sensitive to column temperature. Our results indicate that their separation improves at lower column temperatures (Figure 2C,F). Similarly, reduced temperatures enhanced the resolution of 5,6α‐ and 5,6β‐ECs (5,6α‐/5,6β‐EC), particularly on the C18 column (Figures 2C,F and 3C,F). The separation of 25‐OHC, 27‐OHC, and 24(*R*)/(*S*)‐OHC posed significant chromatographic challenges on reversed‐phase columns. Lower column temperatures improved the separation of 25‐OHC from the other oxysterols in the ACN system (Figure 2C,F). Likewise, reduced temperatures slightly enhanced the separation of 27‐OHC in the MeOH system (Figure 3C,F). However, under all tested conditions, 24*S*‐ and 27‐OHC remained unresolved in the ACN mobile phase, while 24*S*‐ and 25‐OHC could not be separated in the MeOH system. Computation (Figure S2) suggests that the differential hydrogen bonding of different analytes may contribute to the observed effects of column separation temperature on selectivity. The hydroxy group in 7α‐OHC is axial and sterically occluded, while the hydroxy group in 7β‐OHC is equatorial and less occluded. The epoxy group in 5,6β‐EC is sterically occluded by a nearby methyl group, while the epoxy group in 5,6α‐EC is less occluded. The less occluded species 7β‐OHC and 5,6α‐EC are more available to form hydrogen bonds with the mobile phase. Likewise, the primary alcohol 27‐OHC is more hydrogen‐bonding than the tertiary alcohol 25‐OHC. Lowering the column temperature increases the strength of hydrogen bonds to the mobile phase, presumably improving the resolution of isomers possessing small differences in hydrogen bonding. Consistent with this hypothesis, resolving the secondary alcohols 24(*R*)/(*S*)‐OHC from the tertiary and primary alcohols 25‐OHC and 27‐OHC remains challenging even at low temperature. The overall RT and partitioning between stationary and mobile phases depend on many factors; however, the steric accessibility of hydrogen bonding sites provides a specific mechanistic contribution to temperature‐dependent selectivity.

#### 3.1.3 Solvent System Selectivity

ACN and MeOH are the most frequently used organic solvents in RPLC, but their different polarities can lead to distinct separation patterns for the same analytes. The primary factor is the solvent's “elution strength,” which describes its ability to remove analytes from the stationary phase; a stronger solvent will elute analytes faster. ACN is generally considered a stronger eluting solvent than MeOH, meaning it will elute analytes faster at the same concentration. The solvent system had a relatively minor effect on the separation of the sterol ring oxidized cholesterol 7α‐/7β‐OHC and 5,6α‐/5,6β‐EC isomer pairs, with MeOH (Figure 3A–F) providing slightly better resolution for both pairs compared to ACN (Figure 2A–F). In contrast, the separation of side chain oxidized cholesterols, 24*R*‐OHC, 24*S*‐OHC, 25‐OHC, and 27‐OHC, was found to be solvent‐dependent. When using ACN as the mobile phase, the elution order was 25‐OHC, 24*S*‐/27‐OHC (unresolved), and 24*R*‐OHC, with the C18 column offering better resolution than C8 (Figure 2C,F). However, when MeOH was used, the elution order shifted to 24*S*‐/25‐OHC (unresolved), 24*R*‐OHC, and 27‐OHC on both columns.

3.7.4| Summary Overall, a lower column temperature at 25°C provided better separation for most of the oxysterols that we have studied. At this column temperature, we achieved the best separation of 7α‐ and 7β‐OHC on a C8 column in an ACN‐based mobile phase (Figure 2C). The isomeric epoxy cholesterols 5,6α‐ and 5,6β‐EC were very well resolved on a C18 column at this temperature using either MeOH (Figure 3F) or ACN (Figure 2F) as the mobile phase. Separation of 24*R*‐OHC, 24*S*‐OHC, 25‐OHC, and 27‐OHC was optimal under two conditions at a lower column temperature of 25°C: C18 with ACN and C8 with MeOH. However, despite the various efforts of optimization, some analytes could not be fully resolved, such as 24*S*‐OHC and 27‐OHC. Fortunately, they were distinguishable based on their unique MRM transitions (385.4 > 367.5 for 24*S*‐OHCand 385.4 > 161.3 for 27‐OHC), enabling accurate individual quantification. In contrast, 24*S*‐OHC and 25‐OHC were difficult to separate in MeOH and shared the same MRM transitions, making their independent quantification more challenging when MeOH is adopted as the mobile phase. Interestingly, 24*R*‐OHC and 27‐OHC were better separated on the C8 column compared to the C18, emphasizing the importance of stationary phase selection in oxysterol analysis.

Together, these findings highlight the critical role of optimizing column selectivity, column temperature, and mobile phase composition in achieving reliable separation of oxysterol isomers using reversed‐phase HPLC. Such optimization is essential for accurate quantification in biological samples.

### 3.2 Oxysterol Levels in Brains of Reference‐Diet and High‐Fat Diet Mice

The C3H mouse strain is a robust model for studying obesity in a long‐term chronic model for age‐related, DIO [16]. This strain offers a valuable alternative model for investigating the growing obesity epidemic in Western societies, especially in populations not genetically predisposed to obesity. As a longevity model, it also provides unique insight into diet‐induced lifestyle factors that contribute to obesity and aging factors often overlooked in transgenic or obesity‐susceptible strains. Furthermore, this mouse model establishes a strong foundation for examining the effects of diet and DIO on aging, particularly in the brain. It also enables exploration of how obesity and diabetes may increase the risk of neurodegenerative diseases, such as Alzheimer's disease.

**FIGURE 4:**
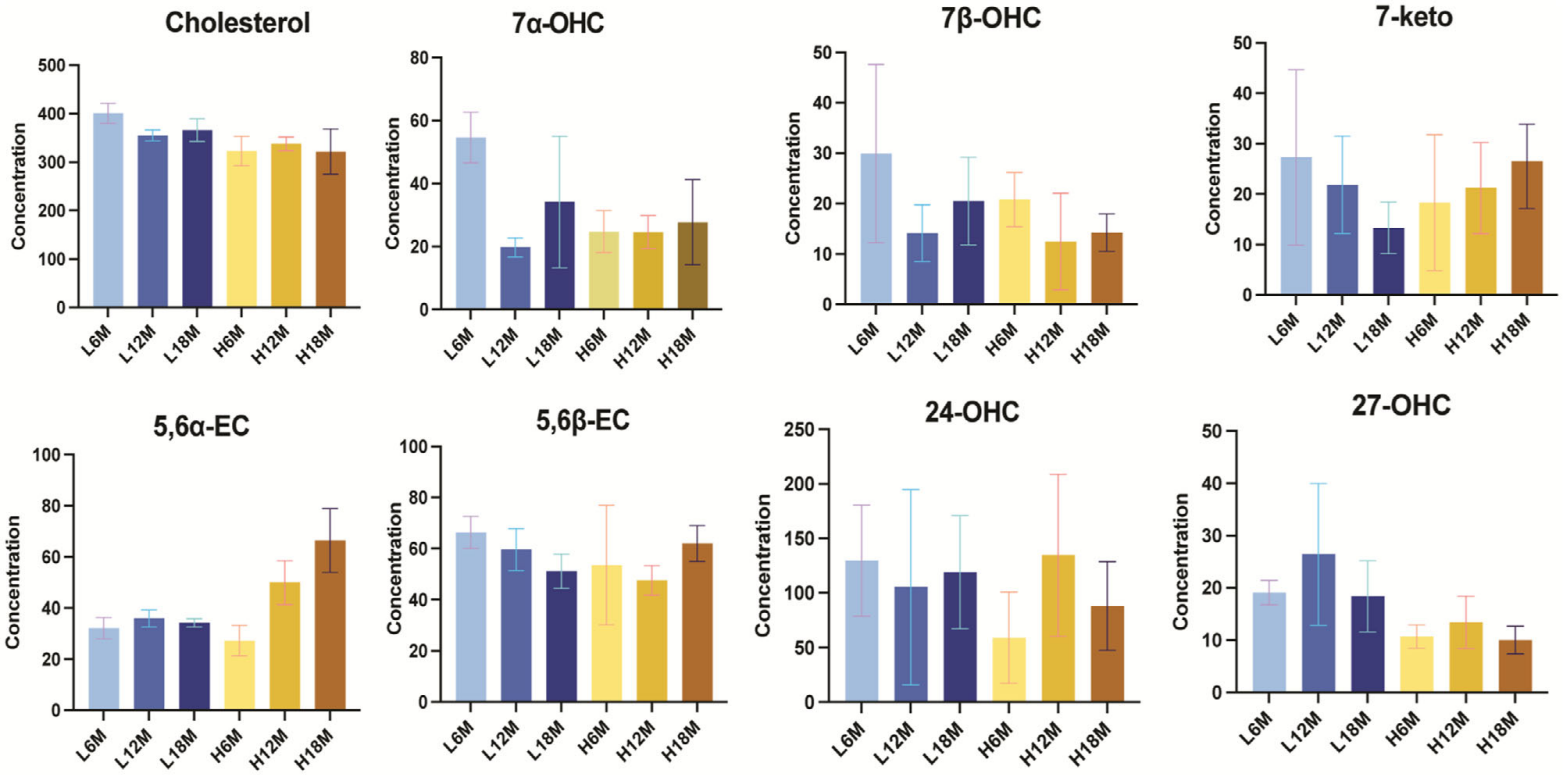
Liver cholesterol and oxysterol concentration. Note the changes in concentrations for the oxysterols as demonstrated by changes in the *Y*‐axis scales. L6M, L12M, and L18M represent the reference diet 6‐, 12‐, and 18‐month‐old mice livers; H6M, H12M, and H18M represent the high‐fat diet 6‐, 12‐, and 18‐month‐old mice liver.

**FIGURE 5:**
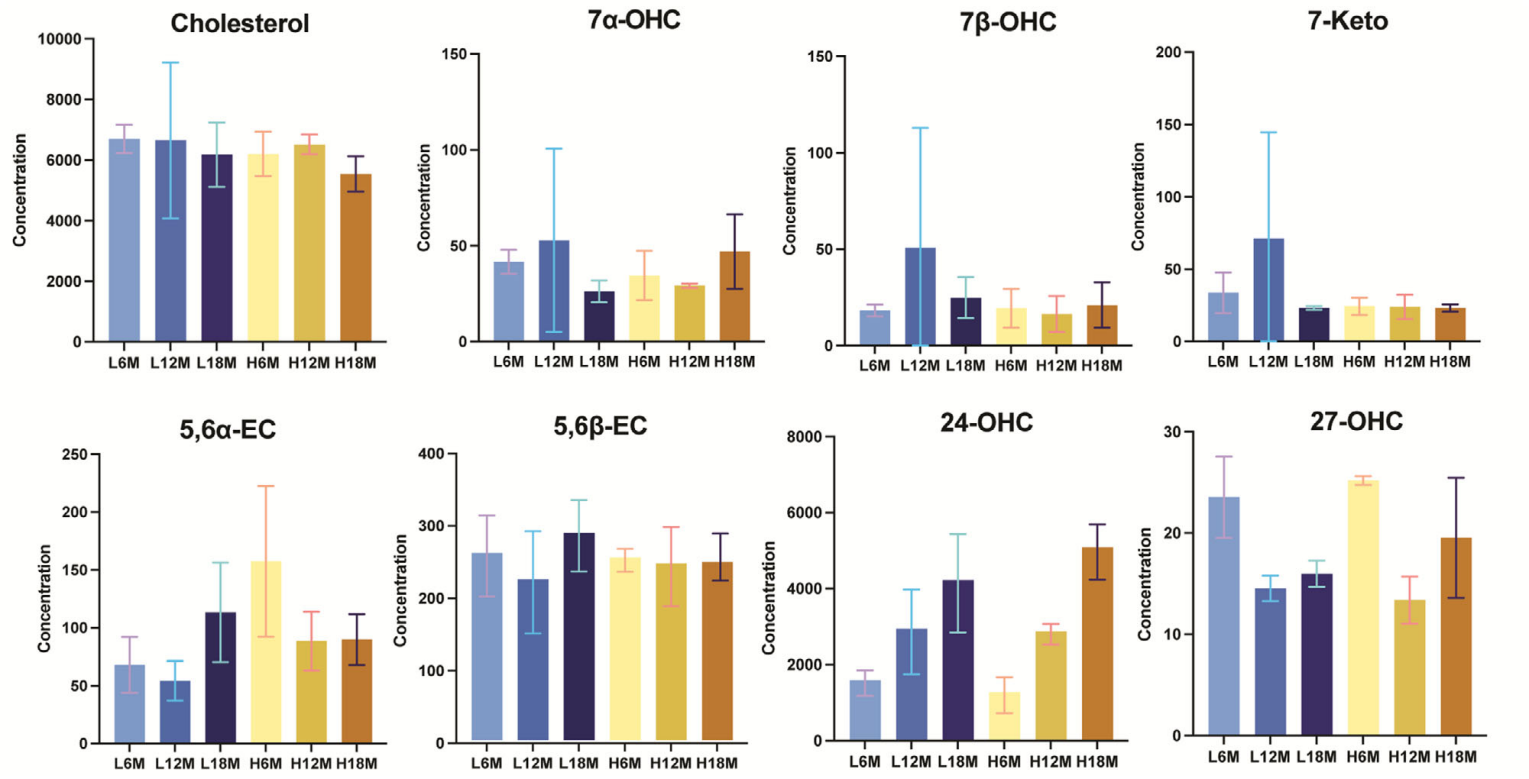
Brain cholesterol and oxysterol concentration. Note the changes in concentrations for the oxysterols as demonstrated by changes in the *Y*‐axis scales. L6M, L12M, and L18M represent the reference diet 6‐, 12‐, and 18‐month‐old mice brains; H6M, H12M, and H18M represent the high‐fat diet 6‐, 12‐, 18‐month‐old mice brains.

In the study by Barr and Gollahon [16], 64 female C3H mice were divided into two groups (*n* = 32) and maintained on either a control diet or a high‐fat diet (HFD) for up to 18 months. At 6‐month intervals, a cross‐sectional cohort (*n* = 8) was selected for evaluation. The mice were monitored for changes in total body mass, lean mass, fat mass, survivability, and tumor incidence. The C3H strain developed DIO, with increased total mass observed in HFD‐fed mice. However, female mice showed a survivability rate of approximately 40%, regardless of diet. In this study, three replicates of female mice from each age and diet group were selected to evaluate cholesterol and oxysterol levels in both liver (Figure 4) and brain tissues (Figure 5).

Based on our optimized LC‐MS method measurement to achieve the best separation of all eight oxysterol isomers at a low column temperature of 25°C, cholesterol levels in the liver and brain remained relatively consistent across different ages and diet groups, showing small variations. However, cholesterol levels detected in the brain are approximately 10 times higher than in the liver. In contrast, the levels of 7α‐OHC, 7β‐OHC, 7‐KC, and 27‐OHC were comparable between brain and liver tissues. Slight variations were observed across different age and diet groups; however, these differences were not statistically significant (Figure 4 and 5).

24*S*‐OHC, a cholesterol metabolite predominantly produced in the brain, exhibited significantly higher concentrations in brain tissue (20–40 times greater compared to the liver). Its levels demonstrated an approximately linear increase with age, independent of dietary influence (Figure 5). 24*S*‐OHC is primarily produced by neurons via the enzyme CYP46A1, which helps regulate cholesterol homeostasis in the brain. Unlike cholesterol, which does not cross the BBB easily, 24*S*‐OHC can exit the brain through the BBB and enter circulation for liver metabolism. The increase in 24*S*‐OHC with age in the brain might be primarily due to age‐related changes in cholesterol metabolism. In contrast, the level of 24*S*‐OHC in the liver detected does not clearly indicate any influence from age or diet. Instead, it reflected a balance between its production in the brain and its rate of elimination by the liver.

Additionally, 5,6α‐EC levels in the brain decreased with increasing age only if the mice were on a high‐fat diet. In contrast, in the liver, 5,6α‐EC level increased with age only on high‐fat diet, thus it influenced by both age and dietary factors

## 4 Conclusion

At a low column temperature of 25°C, 7α‐ and 7β‐OHC achieved the best separation on both the C8 and C18 columns using an ACN‐based mobile phase, while 5,6α‐ and 5,6β‐EC were well resolved on a C18 using either an ACN‐ or MeOH‐based mobile phase. Optimal separation of 24*R*‐OHC, 24*S*‐OHC, 25‐OHC, and 27‐OHC was observed at this temperature as well using a C18 column with ACN or using a C8 column with MeOH as mobile phases. With the same low column temperature at 25°C, we used a C18 column and ACN as a mobile phase to quantify cholesterol and eight oxysterols in mice undergoing a longitudinal study on control and high‐fat diets. 24*S*‐OHC showed a robust linear correlation with age in both diet groups, suggesting its potential as an aging biomarker for adult mice. Whereas 5,6α‐epoxycholesterol levels were influenced by both age and dietary factors, showing an inverse correlation with age in mice on a high‐fat diet.

The follow‐up computational prediction of oxysterol chromatographic behavior, based on current examples, will be expanded to a broader range of lipids using machine‐learned RT models. This approach has the potential to advance de novo RT prediction across various chromatographic methods and, in turn, guide more effective optimization of separation conditions.

## Author Contributions

**Indhumathy Subramaniyan**: conceptualization, methodology, writing – review and editing. **Benjamin Barr**: resources, writing – review and editing. **Ninh M. La‐Beck**: conceptualization, writing – review and editing. **Benjamin G. Janesko**: methodology, writing – review and editing. **Lauren Gollahon**: resources, conceptualization, writing – review and editing. **Li**: conceptualization, methodology, writing – original draft, writing – review and editing.

## Conflicts of Interest

The authors declare no conflicts of interest.

## Supporting information




**Supporting Information file 1**: jssc70274‐sup‐0001‐SuppMat.docx

## Data Availability

All data are available in the main text or the Supporting Information.
