# Supplementary material for: Identifying Oxysterols Associated With Age and Diet in Mice Using Optimized Reversed‐phase Liquid Chromatography‐Mass Spectrometry (RPLC‐MS)
Source: J Sep Sci. 2025 Sep 29;48(10):e70274. doi: 10.1002/jssc.70274 (PMC12480644; doi:10.1002/jssc.70274)
Supplement: Supplementary file 1 — Supporting Information file 1: jssc70274‐sup‐0001‐SuppMat.docx [file JSSC-48-e70274-s001.docx]

*Supplementary Information:*

**Identifying Oxysterols Associated with Age and Diet in Mice Using Optimized Reverse Phase Liquid Chromatography-Mass Spectrometry (RPLC-MS)**

Indhumathy Subramaniyan^1^, Benjamin Barr^2^, Ninh M. La-Beck^3^, Benjamin G. Janesko^4^, Lauren Gollahon*^2^ , Li Li*^1^

1. Clinical Pharmacology and Experimental Therapeutics Center, Jerry H. Hodge School of Pharmacy, Texas Tech University Health Sciences Center, 5920 Forest Park RD. Dallas, TX 75235, USA;

2. Department of Biological Sciences, Texas Tech University, 2500 Broadway, Lubbock, TX 79409, USA;

3. Department of Immunotherapeutics and Biotechnology, Texas Tech University Health Sciences Center, Jerry H. Hodge School of Pharmacy, Abilene, Texas, USA

4. Department of Chemistry & Biochemistry, Texas Christian University, Fort Worth, TX 76129, USA

* Corresponding author: li.li@ttuhsc.edu

**Table S1. Cholesterol and oxysterol precursors and MRM pairs.** Major MRMs used for quantitative analysis were noted in black, minor MRMs were noted in blue.

| **Analyte** | **Abbreviation** | **MS scan (+)** | **MRM pairs (+)** | **DP** | **CX** | **CXP** |
| --- | --- | --- | --- | --- | --- | --- |
| 25-hydroxycholesterol | **25-OHC** | 385.4 | 385.4>367.5 | 65 | 12 | 20 |
|  |  | 367.4 | 367.4>147.1 | 105 | 30 | 15 |
|  |  | 367.4 | 367.4>158.5 | 105 | 30 | 15 |
| 24(S)-hydroxycholesterol | **24(S)-OHC** | 385.4/367.4 | 385.4>367.5 | 60 | 14 | 16 |
|  |  | 367.4 | 367.4>95.0 | 105 | 30 | 15 |
|  |  | 367.4 | 367.4>147.1 | 105 | 30 | 15 |
| 24(R)-hydroxycholesterol | **24(R)-OHC** | 385.4 | 385.4>367.5 | 75 | 25 | 20 |
| 27-hydroxycholesterol | **27-OHC** | 385.4 | 385.4>161.3 | 75 | 15 | 12 |
|  |  | 385.4 | 385.4>81.1 | 105 | 30 | 15 |
|  |  | 385.4 | 385.4>95.1 | 105 | 30 | 15 |
| 5,6-⍺-epoxycholesterol | **5,6⍺-OHC** | 403/385/367 | 385.4>367.1 | 95 | 20 | 15 |
|  |  | 385.4 | 385.4>95.1 | 80 | 33 | 10 |
| 5,6-β-epoxycholesterol | **5,6β-OHC** | 385.4 | 385.4>367.3 | 95 | 20 | 15 |
|  |  | 385.4 | 385.4>95.1 | 70 | 32 | 8 |
| 7-Ketocholesterol | **7-keto** | 401.4 | 401.4>383.3 | 105 | 29 | 13 |
|  |  | 401.4 | 401.4>95.1 | 185 | 30 | 18 |
|  |  | 401.4 | 401.4>105.5 | 185 | 30 | 18 |
| 7β-hydroxyholesterol | **7β-OHC** | 385/367 | 385.4>367.5 | 100 | 20 | 16 |
|  |  | 367.4 | 367.4>147.1 | 70 | 32 | 8 |
|  |  | 367.4 | 367.4>159.1 | 70 | 32 | 8 |
| 7⍺-hydroxycholesterol | **7⍺-OHC** | 385.4 | 385.4>367.5 | 60 | 15 | 27 |
|  |  | 367.4 | 367.4>147.1 | 80 | 33 | 10 |
|  |  | 367.4 | 367.4>159.1 | 80 | 33 | 10 |
| Cholesterol | **Cholesterol** | 369.3 | 369.3>161.1 | 128 | 17 | 20 |
|  |  | 369.3 | 369.3>147.2 | 185 | 28 | 9 |

**Table S2. Predicted Retention Times.** The computed hexane:water partition coefficients logP_HW_ are compared to the measured RT (Figure 2C and 2F, 25^o^C). Correlation coefficients R^2^ between the oxysterols’ measured RT and computed logP_HW_ are included. All computed water-to-hexane transfer free energies are positive, indicating that the species are predicted more soluble in hexane and less soluble in water.

|  | RT (s), C8, 25^o^C | RT (s), C18, 25^o^C | logP_HW_ |
| --- | --- | --- | --- |
| 7α-hydroxycholesterol | 4.1 | 10.2 | 4.35 |
| 7β-hydroxycholesterol | 3.8 | 10.6 | 3.83 |
| 7-ketocholesterol | 4.15 | 11.1 | 3.79 |
| 5,6α-epoxycholesterol | 5.4 | 14.5 | 4.89 |
| 5,6β-epoxycholesterol | 5.1 | 14 | 4.93 |
| 24(R)-hydroxy cholesterol | 2.6 | 5.9 | 3.70 |
| 24(S)-hydroxy cholesterol | 2.4 | 5.8 | 3.65 |
| 25-hydroxy cholesterol | 2.2 | 5.2 | 3.29 |
| 27-hydroxycholesterol | 2.4 | 5.8 | 3.26 |
| Cholesterol |  |  | 7.22 |
| Predicted:Experiment R^2^ | 0.85 | 0.81 |  |

**Figure S1. Extraction recovery rate and matrix effect.** The extraction recovery of cholesterol and its oxysterols at 100ng/mL were determined by comparing the peak area response of analytes from extracted samples (n=6) with the peak area response of analytes from post-extraction spiked tissue homogenate samples. The percent recovery was calculated by taking the ratio of the analytes’ peak area of extracted samples (i.e. post extracted spiked samples) to the peak area of the post extracted spiked samples. Matrix effect was assessed by taking the ratio of the analytes’ peak area in the presence of matrix (i.e. post extracted spiked samples) to the peak area in the absence of matrix (i.e. pure solution of analytes). Matrix factor=1 means no matrix effect, <1 suggests ion suppression and a value > 1 suggests ionization enhancement.

7β-OHC, equatorial

7α-OHC, axial & occluded


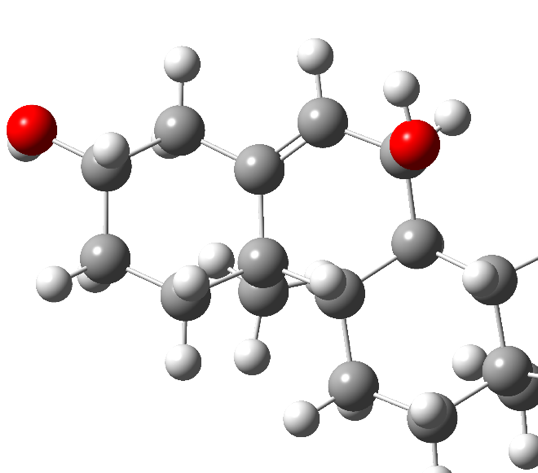

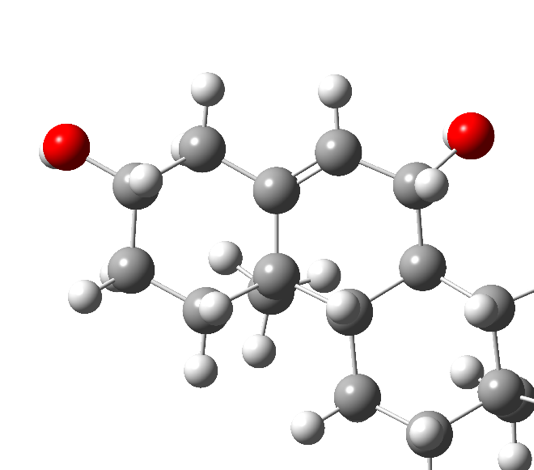


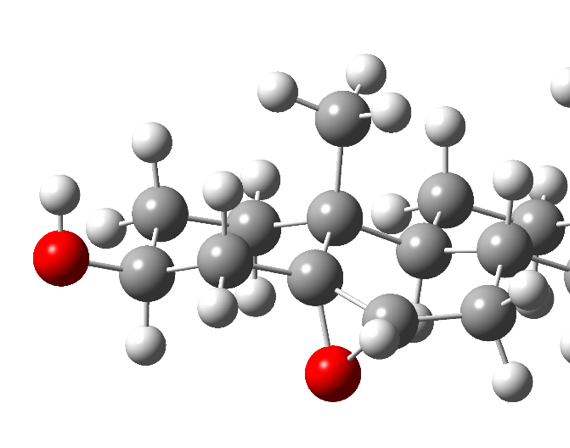

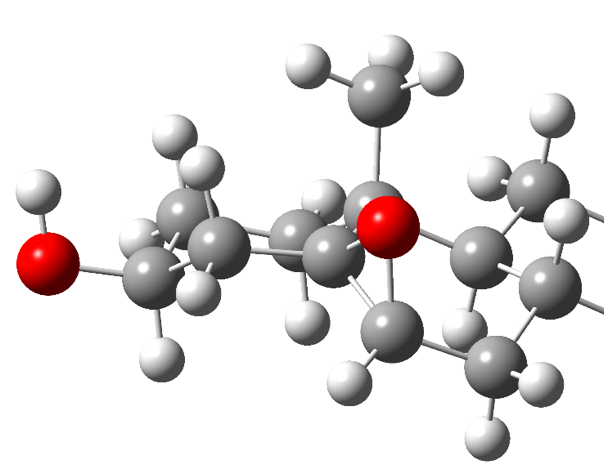


5,6α-OC, opposite face to methyl

5,6β-OC, same face to methyl & occluded

**Figure S2.** DFT-predicted structures of selected oxysterol isomer pairs, highlighting the steric occlusion of hydrogen-bonding groups.
